# *﻿Notalina* (*Neonotalina*) *﻿ralphi* sp. nov. (Trichoptera, Leptoceridae), a new long-horned caddisfly from the Cerrado biome of Brazil, with new records for *﻿N.* (*﻿Neonotalina*) *﻿brasiliana* Holzenthal, 1986 and an identification key

**DOI:** 10.3897/zookeys.1111.77581

**Published:** 2022-07-11

**Authors:** Erica Silva Pereira, Ian Oliveira, Gleison Robson Desidério, Adolfo Calor, Neusa Hamada

**Affiliations:** 1 Programa de Iniciação Científica, Instituto Nacional de Pesquisas da Amazônia (INPA), Manaus, Amazonas, Brazil Instituto Nacional de Pesquisas da Amazônia (INPA), Coordenação de Pós-Graduação (COPOG) Manaus Brazil; 2 Laboratório de Entomologia Aquática, Departamento de Zoologia, Instituto de Biologia, Universidade Federal da Bahia, Rua Barão de Geremoabo, 147, campus Ondina, CEP 40170-290, Salvador, Bahia, Brazil Programa de Iniciação Científica, Instituto Nacional de Pesquisas da Amazônia (INPA) Manaus Brazil; 3 Instituto Nacional de Pesquisas da Amazônia (INPA), Coordenação de Pós-Graduação (COPOG), Divisão do Curso em Entomologia (DiEnt), Coordenação de Biodiversidade (CoBio), Manaus, Amazonas, Brazil Universidade Federal da Bahia Salvador Brazil

**Keywords:** Aquatic insects, geographic distribution, taxonomy, Triplectidinae

## Abstract

The long-horned caddisfly genus *Notalina* Mosely, 1936 contains 27 species divided into two subgenera. The Neotropical N. (Neonotalina) Holzenthal, 1986 occurs exclusively in South America. Its species are organized into two species groups, *brasiliana* and *roraima*. Nine species have been recorded so far in Brazil, mainly distributed in the Cerrado and Atlantic Forest biomes of Southeast Region, and only one species has been recorded from the Central-West and Northeast Regions. In this paper a new species of N. (Neonotalina) is described and illustrated based on adult males from two protected and preserved areas in the Cerrado biome of Brazil. Notalina (Neonotalina) ralphi**sp. nov.** belongs to the *brasiliana* species group and can be recognized mainly by the morphology of the preanal appendages and segment X. New distributional records are provided for N. (Neonotalina) brasiliana Holzenthal, 1986. Additionally, a key to identify males of the ten species in the *brasiliana* species group is provided.

## ﻿Introduction

Leptoceridae, or long-horned caddisflies, with ~2,200 species, is the second most species-rich trichopteran family (Morse et al. 2019). Based on the current classification of the family proposed by Malm and Johanson (2011), four subfamilies are recognized: Grumichellinae Morse, 1981, Leptocerinae Leach, 1815, Leptorussinae Morse, 1981, and Triplectidinae Ulmer, 1906.

*Notalina* Mosely, 1936 belongs to Triplectidinae and contains 27 species divided into two subgenera, the nominotypical Notalina (Notalina) (15 species) and Notalina (Neonotalina) Holzenthal, 1986 (12 species) restricted to the Australasian and Neotropical regions, respectively (Calor 2008; Holzenthal and Calor 2017; Henriques-Oliveira et al. 2018). *Notalina* species are easily distinguished in the adult stage, but not in the immature stages (Holzenthal 1986; Calor and Froehlich 2008). Two species groups (*brasiliana* and *roraima*) were informally defined in the Neotropical subgenus Neonotalina by Holzenthal (1986) based mainly on characters of the male genitalia. Later, the monophyly of both the species groups and subgenera were supported by Calor et al. (2006). The *brasiliana* group is characterized by having a complex phallic apparatus with acuminate lateral flanges at the apex and a well-developed phallotremal sclerite, while in *roraima* group, the phallic apparatus is simple with spatulate lateral flanges at the apex and a small phallotremal sclerite (Holzenthal 1986).

In the Neotropical region, N. (Neonotalina) occurs exclusively in South America. Its highest species diversity occurs in Brazil, with nine species described (*N.brasiliana* Holzenthal, 1986, *N.cipo* Holzenthal, 1986, *N.franciscana* Henriques-Oliveira, Rocha & Nessimian, 2018, *N.froehlichi* Calor & Holzenthal, 2006, *N.goianensis* Calor, 2008, *N.hamiltoni* Holzenthal, 1986, *N.jordanensis* Henriques-Oliveira, Spies & Dumas, 2012, *N.morsei* Holzenthal, 1986, and *N.paulista* Calor & Holzenthal, 2006), distributed mainly in the highlands of the Cerrado and Atlantic Forest biomes of Southeastern region of the country (Calor and Santos 2021).

In this study, we describe and illustrate a new species of N. (Neonotalina) based on adult males from two protected and preserved areas in the Cerrado biome of Central-west and North regions of Brazil, in Federal District and Tocantins states, respectively. We also provide new distributional records for *N.brasiliana*. In addition, a key is provided to identification of males of species in the *brasiliana* group.

## ﻿Materials and methods

Specimens were collected mainly in streams of three conservation units of the Brazilian Cerrado biome. Two of them located in the Federal District, midwestern Brazil (Estação Ecológica de Águas Emendadas (ESECAE) in Planaltina and Parque Nacional de Brasília (PNB) in Brasília) and the third unit located in the Tocantins state, northern Brazil ( Parque Estadual do Lajeado (PEJ), located in the Palmas municipality). One additional specimen was collected in a river in the São Desidério municipality, west of Bahia state, northeast region. Adults were collected by Malaise trap (Gressit and Gressit 1962) and light traps positioned near and about the water. The specimens were preserved in 80% ethanol.

In order to observe male genital structures, the abdomen of each specimen was removed and diaphanized using heated 10% KOH as detailed by Blahnik and Holzenthal (2004). After diaphanization, the abdomen was mounted with glycerin on a temporary slide and was examined under a Leica DM5500 B compound microscope. After observation, the abdomen was permanently stored in glycerin in a microvial, together with the remainder of the respective specimen in a plastic vial with ethanol (Desiderio et al. 2021).

Photographs of the habitus, head and wings of adults were obtained using a Leica DFC420 video camera attached to a Leica M165C stereomicroscope and with a LED illumination dome (Kawada and Buffington 2016). Photographs of the male genitalia were taken with a Leica DFC295 video camera attached to a Leica DM5500B compound microscope. Stacks of images of each structure were then combined automatically into a single image using Helicon Focus Pro stacking software (version 7.6.4). Stacked images of the genitalia were used as templates in Adobe Illustrator for vector illustrations. All photographs and illustrations were assembled into plates using Adobe Photoshop.

The distribution map was prepared using QGIS Las Palmas 2.18.10 software (QGIS Development Team 2016). Vector and raster maps used IBGE (2019) and Natural Earth (2020) data. Morphological terminology follows Holzenthal (1986) and Calor et al. (2006) for the male genitalia with modifications. The species description and identification key were constructed using the DELTA software (Description Language for Taxonomy) (Dallwitz et al. 1993, 2016). Lists of material examined were prepared using the AUTOMATEX macro in Microsoft Excel (Brown 2013).

Types and other material examined are deposited in the following collections: Coleção de Invertebrados, Instituto Nacional de Pesquisas da Amazônia, Manaus, Brasil (**INPA**), Museu de Zoologia da Universidade Federal da Bahia, Salvador, Brazil (**UFBA**), Museu de Zoologia da Universidade de São Paulo, São Paulo, Brazil (**MZUSP**), University of Minnesota Insect Collection, St. Paul, Minnesota, USA (**UMSP**), Coleção Entomológica Prof. José Alfredo Pinheiro Dutra, Departamento de Zoologia, Universidade Federal do Rio de Janeiro, Rio de Janeiro, Brazil (**DZRJ**), and Coleção Entomológica Padre Jesus Santiago Moure, Departamento de Zoologia, Universidade Federal do Paraná, Curitiba, Brazil (**DZUP**).

## ﻿Taxonomy

### ﻿ Notalina (Neonotalina) ralphi sp. nov.

Taxon classificationAnimaliaTrichopteraLeptoceridae

1FBA42BB-6DC6-56AB-96FB-86C56C2764FE

http://zoobank.org/F6A903EA-547F-4D59-9BE2-EC6232601A22

Figs 1 , 2

#### Diagnosis.

This new species can be easily recognized by the absence of processes on the median portion of tergum X, inferior appendages with ventromesal process indistinct laterally and distinct ventrally with subtruncated apex, and Y-shaped phallotremal sclerite in lateral view. *Notalinaralphi* sp. nov. is morphologically similar to *N.franciscana* based on the subtruncate mesoventral process of the inferior appendages in ventral view. However, *N.ralphi* sp. nov. has the acuminate preanal appendages, which in *N.franciscana* are clavate. In addition, in the new species the ventrolateral margin of the segment X has stout, short setae, whereas *N.franciscana* has these setae only in the apex of the tergum.

#### Description.

**Figure 1.**
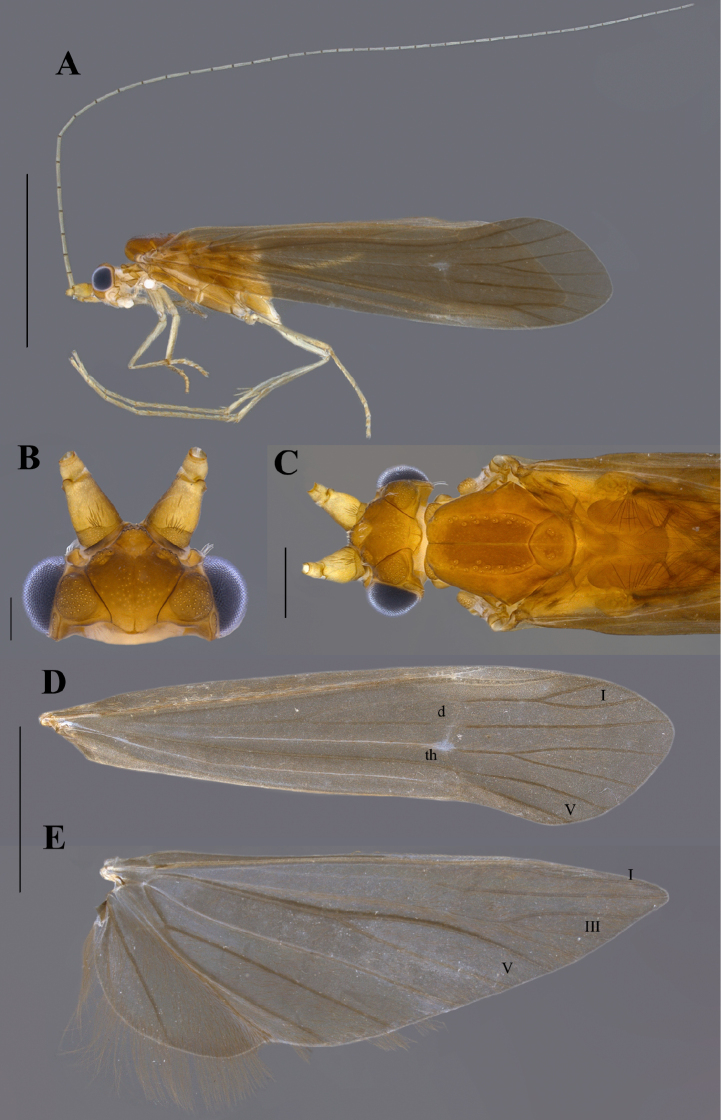
Notalina (Neonotalina) ralphi sp. nov., holotype, ♂ (INPA) **A** lateral habitus **B** head, dorsal view **C** head and thorax, dorsal view **D** forewing, right dorsal view **E** hind wing, right dorsal view. Scale bars: 0.2 mm (**B**); 0.5 mm (**C**); 2 mm (**A, D, E**).

**Figure 2.**
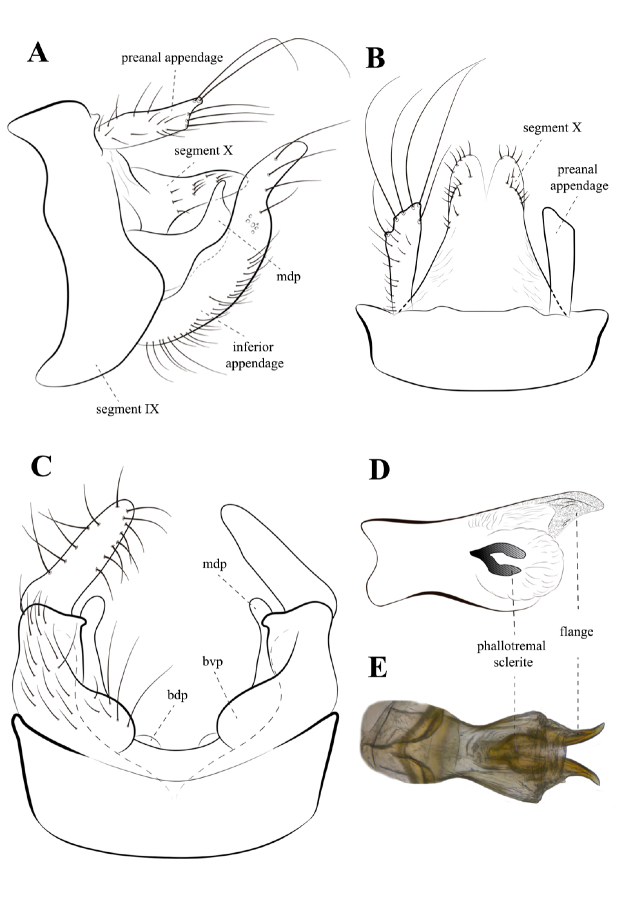
Notalina (Neonotalina) ralphi sp. nov., male genitalia, holotype **A** lateral view **B** dorsal view **C** ventral view **D** phallic apparatus, lateral view **E** phallic apparatus, ventral view. Abbreviations: bdp, basodorsal process; bvp, basoventral process; mdp, mesodorsal process.

**Male.** Head brown (in alcohol) (Fig. 1B); maxillary and labial palps brown; antennae pale brown (Fig. 1A). Thorax brown; pleuron pale brown (Fig. 1C). Forewing brown, with small hyaline spot at thyridial cell; forewing length 7.8 mm (*n* = 4), forks I and V present (Fig. 1D); hind wing length 6 mm (*n* = 4), forks I, III, and V present, fork I very narrow and fork III with very short petiole (Fig. 1E). Legs pale brown; tibial spur formula 2, 2, 4 (Fig. 1A). Segment IX, in lateral view, broadest ventrolaterally, anterior margin slightly sinuous (Fig. 2A); apicodorsal area with paired, poorly developed, distantly situated protuberance; posterolateral margin bearing setae. Preanal appendages setose, long, and slender, ~ 2/3 length of segment X (Fig. 2A, B); in dorsal view, apex acuminated towards inner margin, bearing long setae (Fig. 2B). Segment X, in lateral view, saddle-shaped; anterodorsal area slightly convex; mid-dorsal area without lateral protuberance; distal area without dorsomesal and dorsolateral processes; apicolateral processes rounded, bearing short stout setae (Fig. 2A); in dorsal view, V-shaped apicomesal incision extending anteriorly ~ 1/3 length of segment X; with a row of 5–7 short stout setae subapically. Inferior appendage, in lateral view, with broad basal portion, apical portion elongate, digitate, setose; basodorsal process rounded, smaller than basoventral process; dorsomesal process long and broad, apex acute, directed apicodorsad (Fig. 2A); in ventral view, basoventral process well developed, slightly asymmetrical, rounded, apex directed mesad (Fig. 2C); ventromesal process, in lateral view, indistinct (Fig. 2A); in ventral view, distinct with subtruncated apex (Fig. 2C). Phallic apparatus with a pair of strongly sclerotized, acuminate phallobase flanges, apex directed dorsad (Fig. 2D); phallotremal sclerite well developed, roughly Y-shaped in lateral view (Fig. 2D), with an anteriorly directed projection when viewed ventrally (Fig. 2E).

#### Type material.

***Holotype*** Brazil • ♂; Federal District, Planaltina, Estação Ecológica de Águas Emendadas, Córrego Tabatinga; 15.545361°S, 47.566222°W, 1047 m, 04–24 Apr. 2018, G.R. Desidério, C.A. Campos, F. Camelo legs.; Malaise trap; INPA.

***Paratypes*** Brazil• 3 ♂♂; same data as for holotype (UMSP) • 4 ♂♂; Federal District, Planaltina, Estação Ecológica de Águas Emendadas, Córrego Tabatinga; 15.545361°S, 47.566222°W; 1047 m a.s.l.; 07–24 Apr. 2018; G.R. Desidério, C.A. Campos, F. Camelo legs; INPA; • 4 ♂♂; same collection data as for preceding; 24 Apr. – 07 May. 2018; UFBA; • 1 ♂; same collection data as for preceding; MZUSP; • 10 ♂♂; Tocantins, Palmas, Parque Estadual do Lajeado, Igarapé da Onça; 10.112361°S, 48.258639°W; 596 m a.s.l.; 06–11 May. 2017; N. Hamada, G. Amora legs; INPA; • 20 ♂♂; same collection data as for preceding; 19 Dec. 2017; INPA; • 16 ♂♂; same collection data as for preceding; UFBA; • 5 ♂♂; same collection data as for preceding; DZRJ; • 5 ♂♂; MZUSP; • 7 ♂♂; same collection data as for preceding; 18 Jan. 2018; DZUP.

#### Etymology.

The new species is named in honor of Dr. Ralph W. Holzenthal (University of Minnesota, USA) in recognition of his efforts to the advancement of the knowledge on Neotropical caddisflies and his contributions in supervising new entomologists.

#### Distribution.

**Figure 3.**
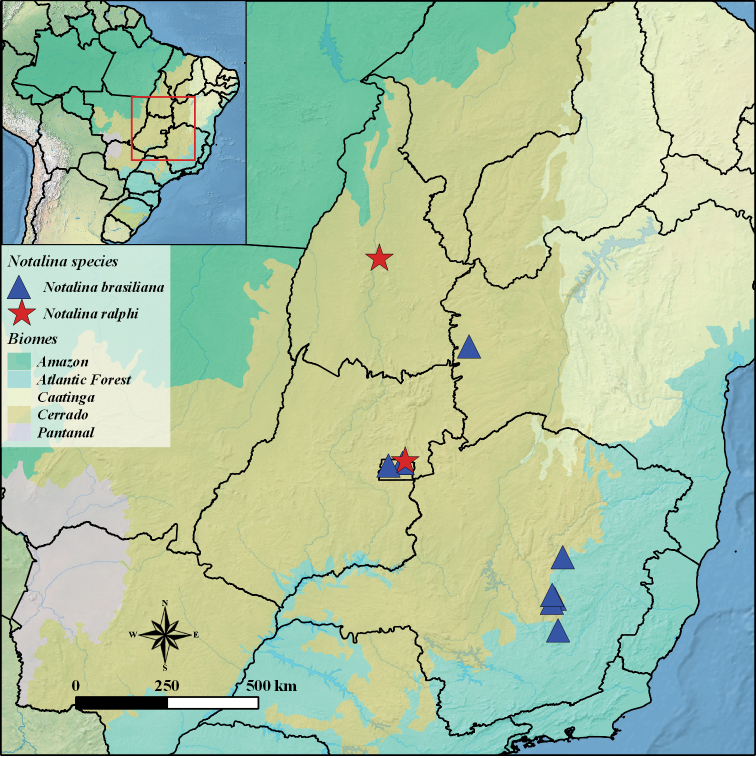
Geographical distribution map of Notalina (Neonotalina) brasiliana Holzenthal, 1986 and Notalina (Neonotalina) ralphi sp. nov.

Brazil: Cerrado biome (Federal District and Tocantins states) (Fig. 3).

##### ﻿New distribution record

### ﻿ Notalina (Neonotalina) brasiliana

Taxon classificationAnimaliaTrichopteraLeptoceridae

Holzenthal, 1986

FDEAFF98-A8EA-5183-9B63-6FA9B7AB061C

Notalina (Neonotalina) brasiliana Holzenthal, 1986: 63 [type locality: Brazil, Minas Gerais, Serra do Caraça; MZUSP; ♂; ♀]; Paprocki et al. 2004: 13 [checklist]; Calor et al. 2006: 41 [distribution]; Paprocki and França 2014: 60 [checklist].

#### Material examined.

Brazil – **Bahia** • 1 ♂; São Desidério, Rio das Fêmeas, BR-020, ponte (#02); 12.466667°S, 45.854583°W; 744 m a.s.l.; 23 Oct. 2008; N. Hamada, G. Fleck, C.A.S. Azevêdo, R. Kikuchi legs; INPA; – **Distrito Federal** • 1 ♂; Planaltina, Estação Ecológica de Águas Emendadas, Córrego Brejinho; 15.592583°S, 47.637333°W; 983 m a.s.l.; 04 Apr. – 24 May. 2018; G.R. Desidério, C.A. Campos, F. Camelo legs; INPA; • 4 ♂♂; Brasília, Parque Nacional de Brasília, Córrego Milho Cozido; 15.662500°S, 48.016556°W; 1076 m a.s.l.; 09 Apr. – 04 Jul. 2018; G.R. Desidério, C.A. Campos, F. Camelo legs; INPA.

#### Distribution.

Brazil: *Cerrado* (Bahia [new record], Distrito Federal [new record] and Minas Gerais States) (Fig. 3).

### ﻿Key to males of Notalina (Neonotalina) brasiliana species group

**Table:** 

1	Preanal appendage long, ~ 2/3 length of segment X (Holzenthal 1986: fig. 3B)	**2**
–	Preanal appendage short, ~ 1/2 length of segment X (Holzenthal 1986: figs 5B, 7B).	**6**
2	Apicolateral processes of segment X mound-like, broad (Fig. 2B)	**3**
–	Apicolateral processes of segment X digitate, slender (Calor 2008: fig. 2E)	**5**
3	Basal portion of inferior appendage slender in lateral view (Henriques-Oliveira et al. 2018: fig. 2A), ventromesal process roughly triangulate in lateral view (Henriques-Oliveira et al. 2018: fig. 2E)	***N.franciscana***
–	Basal portion of inferior appendage broad in lateral view (Fig. 2A), ventromesal process rounded or inconspicuous in lateral view (Fig. 2D)	**4**
4	Preanal appendages acuminate (Fig. 2B); segment IX broadest ventrolaterally (Fig. 2A); submedian area of segment X without lateral protuberance, distal area without dorsomesal processes (Fig. 2B); dorsomesal process of inferior appendage broad (Fig. 2A)	***N.ralphi* sp. nov.**
–	Preanal appendages digitate (Henriques-Oliveira et al. 2012: fig. 3); segment IX broadest laterally (Henriques-Oliveira et al. 2012: fig. 2); submedian area of segment X with lateral protuberance, distal area with dorsomesal processes (Henriques-Oliveira et al. 2012: figs 2, 3); dorsomesal process of inferior appendage slender (Henriques-Oliveira et al. 2012: fig. 2)	***N.jordanensis***
5	Dorsomesal processes of segment X short, 1/2 the length of the ventrolateral processes (Calor 2008: fig. 2E); apical portion of inferior appendage ca. the same length as basal portion (Calor 2008: fig. 2A); ventromesal process roughly triangulate in lateral view (Calor 2008: fig. 2A); apex of phallotremal sclerite single-pointed (Calor 2008: fig. 2D	***N.goianensis***
–	Dorsomesal processes of segment X long, 1/3 longer than the ventrolateral processes (Holzenthal 1986: fig. 3B); apical portion of inferior appendage longer than basal portion (Holzenthal 1986: fig. 3A); ventromesal process blade-like in lateral view (Holzenthal 1986: fig. 3A); apex of phallotremal sclerite bi-pointed (Holzenthal 1986: fig. 3E)	***N.brasiliana***
6	Apicodorsal area of segment IX with single or paired protuberances (Holzenthal 1986: fig. 8B; Calor et al. 2006: figs 1C, 2C); apicolateral processes of segment X digitate, slender (Calor et al. 2006: figs 1C, 2C)	**7**
–	Apicodorsal area of segment IX without protuberances; apicolateral processes of segment X mound-like, broad (Holzenthal 1986: figs 5B, 7B)	**9**
7	Distal area of segment X with dorsomesal processes (Calor et al. 2006: fig. 1A, C); basal portion of inferior appendage slender (Calor et al. 2006: fig. 1A); basoventral process symmetrical, triangulate (Calor et al. 2006: fig. 1B); phallobase with basodorsal process (Calor et al. 2006: fig. 1D)	***N.froehlichi***
–	Distal area of segment X without dorsomesal processes (Holzenthal 1986: fig. 8B); basal portion of inferior appendage broad (Holzenthal 1986, fig. 8A; Calor et al. 2006: fig. 2A), basoventral process asymmetrical, somewhat truncate (Holzenthal 1986: fig. 8C; Calor et al. 2006: fig. 2B); phallobase without basodorsal process	**8**
8	Apicodorsal area of segment IX with single protuberance (Holzenthal 1986: fig. 8B); segment IX broadest laterally (Holzenthal 1986: fig. 8A); distal area of segment X without dorsolateral processes (Holzenthal 1986: fig. 8A, B); apical portion of inferior appendage shorter than basal portion in lateral view (Holzenthal 1986: fig. 8A); phallotremal sclerite slender, single-pointed (Holzenthal 1986: fig. 8E)	***N.hamiltoni***
–	Apicodorsal area of segment IX with paired protuberances (Calor et al. 2006: fig. 2B); segment IX broadest ventrolaterally (Calor et al. 2006: fig. 2A); distal area of segment X with dorsolateral processes (Calor et al. 2006: fig. 2A, C); apical portion of inferior appendage longer than basal portion in lateral view (Calor et al. 2006: fig. 2A); phallotremal sclerite broad, bipointed (Calor et al. 2006: fig. 2E)	***N.paulista***
9	Distal area of segment X with dorsolateral processes; without dorsomesal processes (Holzenthal 1986: fig. 7A, B); basal portion of inferior appendage broad, with ridge (Holzenthal 1986: fig. 7A), basoventral process symmetrical (Holzenthal 1986: fig. 7C); apex of phallotremal sclerite directed ventrally (Holzenthal 1986: fig. 7D)	***N.cipo***
–	Distal area of segment X without dorsolateral processes; with dorsomesal processes (Holzenthal 1986: fig. 5A, B); basal portion of inferior appendage slender, without ridge (Holzenthal 1986: fig. 5A), basoventral process asymmetrical (Holzenthal 1986: fig. 5C); apex of phallotremal sclerite directed dorsally (Holzenthal 1986: fig. 5D)	***N.morsei***

## ﻿Discussion

The species diversity of N. (Neonotalina) in Brazil is concentrated in the Atlantic Forest and Cerrado biomes of Southeastern region with eight species (*N.brasiliana*, *N.cipo*, *N.franciscana*, *N.froehlichi*, *N.hamiltoni*, *N.jordanensis*, *N.morsei* and *N.paulista*). So far, only one species of N. (Neonotalina) has been recorded from the Central-West (*N.goianensis*) and Northeast (*N.cipo*) regions (Calor 2008; Dias et al. 2015). However, with the discovery of *N.ralphi* sp. nov. described here and the new records of *N.brasiliana*, the number of N. (Neonotalina) species recorded from the Central-West and Northeast regions is increased to two species each, bringing the total number of species of the subgenus for Brazilian Cerrado biome to eight (Table 1).

**Table 1.** Distribution of Notalina (Neonotalina) species recorded from Brazil. Abbreviations for Brazilian states: BA = Bahia; Federal District = DF; ES = Espírito Santo; Goiás = GO; MG = Minas Gerais; RJ = Rio de Janeiro; SP = São Paulo; Tocantins = TO.

Species	Region (state)	Biome
*N.brasiliana* Holzenthal, 1986	Southeast (MG); Central-West (DF); Northeast (BA)	Cerrado
*N.cipo* Holzenthal, 1986	Southeast (MG); Northeast (BA)	Atlantic Forest; Cerrado
*N.franciscana* Henriques-Oliveira, Rocha & Nessimian, 2018	Southeast (MG)	Cerrado
*N.froehlichi* Calor & Holzenthal, 2006	Southeast (MG)	Cerrado
*N.goianensis* Calor, 2008	Central-West (GO)	Cerrado
*N.hamiltoni* Holzenthal, 1986	Southeast (SP)	Atlantic Forest
*N.jordanensis* Henriques-Oliveira, Spies & Dumas, 2012	Southeast (SP)	Atlantic Forest
*N.morsei* Holzenthal, 1986	Southeast (ES, MG, RJ, SP)	Atlantic Forest; Cerrado
*N.paulista* Calor & Holzenthal, 2006	Southeast (MG, SP)	Atlantic Forest; Cerrado
*N.ralphi* sp. nov.	Central-West (DF); North (TO)	Cerrado

The occurrence of N. (Neonotalina) species in the Federal District represents the first record of Integripalpia for the federative unit. Previously, only seven species of Annulipalpia were known (Santos et al. 2021). In addition, the record of *N.ralphi* sp. nov. in Tocantins state is the northernmost record of the *brasiliana* species group, previously established by *N.goianensis* from the Chapada dos Veadeiros, Goiás state (Calor 2008). Notalina (Neonotalina) brasiliana was previously known only from the Serra do Caraça and Serra do Cipó (Holzenthal 1986), two mountainous regions located in the southern portion of the Espinhaço mountain range, in the Minas Gerais state. Calor et al. (2006) reported this species for other mountains of Minas Gerais state, also in Cerrado biome (Parque Estadual do Rio Preto and Serra do Abreu). Here, the distribution range of this species is extended to Cerrado biome in the Bahia state and Federal District, representing the first records for Northeast and Central-West regions of Brazil, respectively.

Notalina (Neonotalina) ralphi sp. nov. has strong affinity to the *brasiliana* species group of Holzenthal (1986) and can be considered a member of this group based on the characteristics of the phallic apparatus. Although its morphological similarities and differences are assessed for the adult stage with *N.franciscana*, the phylogenetic relationships with other species in the *brasiliana* species group should be evaluated under a combined morphological/molecular phylogenetic approach.

Therefore, this study highlights the need for more taxonomic studies focused on N. (Neonotalina) in Brazil, especially in the poorly sampled Amazon, Caatinga, Pampas, and Pantanal biomes, as well as an updated phylogenetic study including species newly described, morphological characters of immature stages, and multi-locus molecular sequence data.

## Supplementary Material

XML Treatment for Notalina (Neonotalina) ralphi

XML Treatment for Notalina (Neonotalina) brasiliana
